# AhR regulation of amyloid beta-induced inflammation in astrocyte cells

**DOI:** 10.3389/fncel.2025.1618209

**Published:** 2025-07-08

**Authors:** Emmanuel Ojo, Temitope Adu, Raheem F. H. AI Aameri, Shelley A. Tischkau

**Affiliations:** ^1^Department of Pharmacology, Southern Illinois University School of Medicine, Springfield, IL, United States; ^2^Department of Medical Microbiology, Immunology and Cell Biology, Southern Illinois University School of Medicine, Springfield, IL, United States

**Keywords:** Alzheimer’s Disease (AD), amyloid beta (Aβ), astrocytes, inflammation, cytokines, immune response, neurotoxic phenotype

## Abstract

**Introduction:**

Amyloid beta (Aβ) plaques, tau tangles, and neuroinflammation are common features present in Alzheimer’s Disease (AD), and glial cells are essential mediators of the inflammatory reaction. Aryl hydrocarbon receptor (AhR), a transcription factor engaged in regulation of immune function, may be involved in the pathogenesis of AD, through modulation of neuroinflammation. This study explores how AhR affects astrocyte function in response to inflammatory stimuli, with emphasis on Aβ.

**Methods and Results:**

In primary hippocampal astrocyte cultures from wild type (WT, C57BL6/J) or AhR germline knockout (AhRKO) mice, pretreatment with the AhR agonist, 6-Formylindolo[3,2-b] carbazole (FICZ), attenuated Aβ-induction of reactive astrocyte development, characterized by decreased astrocyte complement C3 expression and decreased proinflammatory cytokine release. In addition, Aβ exposure exacerbated TNF-a cytokine release and increased GFAP immunoreactivity in astrocytes derived from AhRKO mice. In response to Aβ injection into the mouse hippocampus *in vivo*, AhRKO mice demonstrated increased astrocyte hypertrophy, reinforcing AhR function in regulating astrocyte responses to neuroinflammation.

**Discussion:**

These findings suggest that AhR activation in astrocytes attenuates development of the neuroinflammatory state, and identifies AhR as an interesting therapeutic target to mitigate neuroinflammation and the progression of AD.

## 1 Introduction

Primary pathological hallmarks for Alzheimer’s Disease (AD) include excessive accumulation of amyloid beta (Aβ) plaques, formation of neurofibrillary tangles, and neuronal loss (Murphy and LeVine, 2010; Hardy and Higgins, 1992). Despite an emerging understanding of the disease’s molecular, biochemical and cellular causes, availability of drugs that can alter disease progression are limited, due to significant adverse effects, high costs, and inadequate effectiveness (Guo et al., 2020; Tatulian, 2022). Although most of the drugs in development over the last decade target Aβ and tau protein, therapeutic approaches that target immune cells within the brain during AD progression are attracting attention (Yiannopoulou and Papageorgiou, 2020; Salomone et al., 2012).

Both innate and adaptive immune responses contribute significantly to AD pathogenesis, and activation of glial cells is a common feature. Activated glial cells release inflammatory molecules, such as cytokines and chemokines, around senile plaques and damaged neurons. Astrocytes are a subtype of glial cells in the brain that are immune-competent, which undergo morphological, functional and molecular changes, in response to endogenous and exogenous stimuli, such as Aβ, cytokines and environmental chemicals (Hyvärinen et al., 2019; Wheeler et al., 2019; Zhao et al., 2021), thereby contributing significantly to the neuroinflammation and neuronal dysfunction observed in mouse models and patients (Matyash and Kettenmann, 2010; Baldwin et al., 2024; Escartin et al., 2019). In human patients and animal models of AD, the disease-associated astrocytes dominate over the homeostatic astrocytes, and promote neurodegeneration (Sochocka et al., 2017; Habib et al., 2020). While cytokines, chemokines and other genes linked to astrocyte inflammatory responses have been used as a biomarker to characterize glia reactivity, morphological changes of glial cells are also recognized as a structural feature present in disease-related states (Endo et al., 2022; Rowland et al., 2024; Vidal-Itriago et al., 2022). Astrocytes in inflammatory conditions can exhibit hypertrophic features, which includes increased soma size, thickening of main branches, and more branching (Sun et al., 2010; Gomes et al., 2024). Along with a molecular signature, dynamic and heterogeneous morphological changes can define astrocyte reactivity in disease states.

Aryl hydrocarbon-receptor (AhR), a member of the basic helix-loop-helix (bHLH)-PAS superfamily, contributes to numerous physiological functions throughout the brain. AhR mediates dendritic morphogenesis, neural progenitor cell differentiation, and is integral to immune responses (Esser and Rannug, 2015; Gassmann et al., 2010; Latchney et al., 2013). Recently, AhR has been linked to several neurodegenerative diseases, including AD. Activation of AhR, by 6-Formylindolo[3,2-b]carbazole (FICZ) and diosmin, increases endogenous Aβ catabolism in APP/PS1 transgenic mice through increasing neprilysin (NEP), which effectively ameliorates memory deficits (Qian et al., 2021). Indoles, confirmed AhR agonists derived from microbial metabolism, are considerably reduced in both AD patients and APP/PS1 mice. Treatment with these same indoles improves cognitive function in APP/PS1 mice through activation of AhR (Sun et al., 2022). The contribution of AhR to glial cell function and inflammatory processes, as well as its contribution to cellular activities response to Aβ during AD development, remain poorly understood. Because AhR action in physiological or pathological states may be cell-type or context specific, understanding how AhR activity in astrocytes impacts Aβ-induced neuroinflammation is warranted. Therefore, this study examines the relationship between AhR signaling pathways and inflammation in astrocytes using neuroinflammatory experimental models relevant to AD.

## 2 Materials and methods

### 2.1 Animals

Protocols for animal use were approved by the Institutional Animal Care and Use Committee at Southern Illinois University School of Medicine and were conducted in accordance with the Guide for the Care and Use of Laboratory Animals as established by the National Institutes of Health. Experiments employed 9–10 weeks old male C57BL/6J, global AhR null (AhRKO) Bradfield strain (Schmidt et al., 1996) and 20 months old male and female APP/PS1 mice procured from the Jackson Laboratory (Bar Harbor, ME) and bred at Southern Illinois University School of Medicine animal facilities. All animals were group housed and entrained to a control 12:12 h light: dark schedule with food and water provided *ad libitum*.

### 2.2 Chemicals

All chemicals were prepared and stored according to manufacturer recommendations unless otherwise noted. Sodium chloride (Cat: 7647-14-5), Sodium phosphate, Dibasic, Anhydrous (Cat: 7558-79-4), Potassium chloride (Cat: 7447-40-7), Potassium phosphate (Cat: 7778-77-01) were obtained from Sigma-Aldrich Co. (St. Louis, MO), Triton X-100 (Cat: 807426) were obtained from MP Biomedicals (Santa Ana, CA).

### 2.3 Primary cell cultures

Primary hippocampal cell cultures were prepared from postnatal day 0–1 C57BL6/J and AhRKO pups by adopting the method previous described (Gutiérrez-Vázquez and Quintana, 2022). Hippocampi were dissected and dissociated by papain (EC 3.4.22.2; Brain Bits) treatment, followed by trituration with sterile glass pipettes. Hippocampal cells were grown on poly-D-lysine (0.05 mg/ml) coated T-75 flasks that contains NbAstro medium (Brain Bits) (Neurobasal, 10% Horse serum, Glutamax and 1% Penicillin/Streptomycin) at 37°C in humidified 5% CO_2_ for (DIV 10–14). The cell culture medium was changed on the day after seeding and then every 3 days. Upon achieving approximately 90% confluence, adherent astrocytes were detached using Trypsin (0.25%) (Cat: 15050057; Gibco, United States) and subsequently related, maintaining them in serum-free media for an additional 1–2 days prior to exposure to 5 μM oligomeric Aβ (1-42) (AnaSpec; Cat: 20276). The purity of cultured astrocyte cells was determined by staining with polyclonal glial fibrillary acidic protein (GFAP).

### 2.4 Aβ (1-42) peptide preparation

The lyophilized peptide powder was reconstituted by the addition of 1.0% NH_4_OH, followed by dilution with sterile PBS to achieve an approximate concentration of 100 μM. This mixture was incubated for 24 h at 4°C. Subsequently, it was centrifuged at 14,000 rpm for 10 min at 4°C, and the supernatant was collected as the oligomeric Aβ peptide, as previously described by Sondag et al. (2009), Dahlgren et al. (2002).

### 2.5 Intrahippocampal injection of Aβ (1-42)

Male mice aged 9–10 weeks were anesthetized with isoflurane and placed on a stereotaxic frame (David Kopf Instruments). A bilateral craniotomy was performed, and 2 μl of 0.5 μg/μl Aβ (1-42) or PBS was administered into the CA1 region of the hippocampus (AP = −1.70 mm, ML = ± 1.2 mm, DV = 1.53 mm) using a 5–10 μL gas-tight Hamilton syringe operating at a flow rate of 0.4 μL/min. Subsequent to the injection, the needle was left for a further 5 min before being gradually retracted. The skin of the mice was subsequently sealed with surgical staples and mice were observed for full recovery. Histological analyses were performed on treated animals 2 weeks after injection.

### 2.6 RNA extraction and qPCR

The expression 2–2.5 × 10^6^ astrocyte cells per T-25 flask were lysed in Trizol (Fisher Scientific, Hampton, NH, United States) and the aqueous layer was collected following chloroform extraction with RNA isolation protocol. RNA concentrations were measured using the Nanodrop spectrophotometer and cDNA was synthesized. SYBR green-based real-time reverse transcriptase PCR was carried out on Quant-Studio real-time PCR system. Values for genes of interest were normalized using GAPDH as the housekeeping gene and the relative levels of mRNA were determined using the ΔΔCt method. Primers sequence for real time PCR is listed in Supplementary Table 1.

### 2.7 ELISA

The expression 2–2.5 × 10^6^ astrocyte cells per T-25 flask were seeded. Cell culture media from primary hippocampal astrocytes were collected following exposure to Aβ (1-42) ELISA was used to quantify levels of IL-1β (Cat BMS6002; Invitrogen), IL-10 (Cat BMS614; Invitrogen) and TNF-alpha (Cat BMS607-3; Invitrogen) secretion following manufacturer directions.

### 2.8 Immunofluorescence (IF)

A total of 50,000 hippocampal astrocyte cells per 4-well glass slide (1.7 cm^2^ per well) were washed with PBS and subsequently fixed with 4% paraformaldehyde (PFA) for 20 min at room temperature. Cells were permeabilized with PBST (0.1M PBS with 0.25% TritonX-100) three times for 7 min, followed by a 1 h incubation in a solution of 10% normal goat serum and 1% bovine serum albumin (BSA). After incubation, primary antibodies were applied to the cells at 4°C overnight. Cells were rinsed with PBST (0.1M PBS with 0.25% TritonX-100) before incubation with secondary antibodies in the dark at room temperature for 2 h. The cells were subsequently subjected to a final wash three times for 7 min in PBST and were cover slipped with ProLong™ Gold antifade reagent containing DAPI. Staining was examined using confocal microscopy, and quantified with National Institute of Health Image J Software 1.48 (RRID:SCR_003070). For immunoreactivity quantification, a threshold value was set and maintained for all images. The percentage area covered and GFAP mean intensity were then calculated. For astrocyte total area size (μm^2^) quantification, Z-stack images containing five optical slices at an interval of 1 μm using a 40x oil immersion objective (numerical aperture 1.3) with adjusted pinhole size of 0.81 AU on a confocal microscopy system (Zeiss LSM800) at a resolution of 1,024 × 1,024 pixels were taken and a surface was built around each astrocyte stained with GFAP, and astrocyte size was determined. Primary antibodies used included: Chicken polyclonal glial fibrillary acidic protein antibody (1:1000 biosensis Catlog:C-1373-50), Rabbit monoclonal IgG2 anti-C3 (1:100 Invitrogen Catlog:PA5-21349) while secondary antibodies used were Goat anti-chicken IgY H&L (Alexa Fluor^®^ 594) 1:1000, Goat anti-rabbit IgG (H + L) (Alexa Fluor^®^ 488) 1:1000.

For tissue staining, 20 μm hippocampal sections were taken using a cryostat (Model HM525 NX, ThermoFisher Scientific). Serial sections were extracted from every sixth section of the hippocampus. Hippocampal slices were subjected to immunofluorescence using a chicken polyclonal glial fibrillary acidic protein (GFAP) antibody at a dilution of 1:500. Sections were permeabilized in PBST (0.1M PBS with 0.25% Triton X-100) and subsequently washed three times for 10 min each in sodium borohydride in PBS (1 mg/ml) for antigen retrieval. To mitigate non-specific binding, slices were subjected to three washes of 10 min each with PBST and subsequently incubated in a solution of 10% normal goat serum and 1% BSA for 1 h. The slices were subsequently treated overnight in primary antibody under a humidity chamber at 4°C. On the subsequent day, slices were washed four times for 10 min in PBST and incubated with the secondary antibody. Incubate Goat anti-chicken IgY H&L (Alexa Fluor ^®^ 594) at a dilution of 1:1000 for a duration of 2 h. The sections were subsequently subjected to a final wash of four cycles, each lasting 10 min, in PBST and were then coverslipped with ProLong™ Gold antifade reagent containing DAPI. Z-stack images containing five optical slices at an interval of 1 μm using a 40x oil immersion objective (numerical aperture 1.3) with adjusted pinhole size of 0.81 AU on a confocal microscopy system (Zeiss LSM800) at a resolution of 1,024 × 1,024 pixels were obtained, and the morphology of astrocytes was assessed with Imaris software. For astrocyte soma size, 40X z stacks Confocal images were imported into the imaris software and a 3D surface were built around the immunofluorescence z-stacks based on GFAP staining of the cells, the total soma size were then determined and was exported.

### 2.9 RNAscope

Hippocampal sections were obtained via a cryostat (Model HM525 NX, ThermoFisher Scientific). Slides were rinsed with RNase-free PBS for 5 min prior to adhering to the manufacturer’s stated methods (Cat QVT4646B), incubated in Protease QF at 40°C for 10 min, followed by three washes in PBS, each lasting 1 min. Post-washing, mRNA in the tissue was hybridized by incubating with a probe targeting AhR at 40°C for 2 h. Sections were subsequently rinsed three times with the manufacturer’s wash buffer for 2 min, with frequent agitation. Subsequent to washing, the slides were incubated for 30 min at 40°C with the pre-amplifier mix solution, followed by a further 30 min incubation at 40°C with the amplifier mix solution. The slides were subsequently rinsed three times with fresh wash buffer, each for a duration of 2 min. The tissue was then incubated with the labeled probe mix solution for 30 min at 40°C, followed by a final wash with wash buffer for 10 min. Subsequent to RNA labeling, tissue slices were treated overnight at 4°C with a chicken polyclonal primary antibody against glial fibrillary acidic protein (GFAP). On the next day, slices were rinsed four times for 10 min in PBST and incubated with the secondary antibody for 2 h. The slides were subsequently cleaned with PBS and coverslipped using ProLong™ Gold antifade reagent containing DAPI. Z-stack images containing five optical slices at an interval of 1 μm were acquired using a 40x oil immersion objective (numerical aperture 1.3) with adjusted pinhole size of 0.81 AU for the red and green channel, while the blue channel had 0.95 AU on a confocal microscopy system (Zeiss LSM800) at a resolution of 1,024 × 1,024 pixels and imported into Zeiss ZEN 3.8 software to manually count the number of AhR puncta co-labeled with GFAP stain.

### 2.10 Statistical analysis

Prism (GraphPad Software, Inc., La Jolla, CA; RRID:SCR_002798) software was used for statistical analyses. Data are presented as mean ± SEM. Unless otherwise stated, all cell culture experiments were repeated at least three times, from separate dissections. One-way or two-way ANOVA with Tukey’s post-hoc tests were utilized to identify significant differences between groups, where appropriate. Statistical significance was defined as *p* < 0.05.

## 3 Results

### 3.1 Oligomerized Aβ (1-42) induces an inflammatory response in astrocyte cells *in vitro*

**FIGURE 1:**
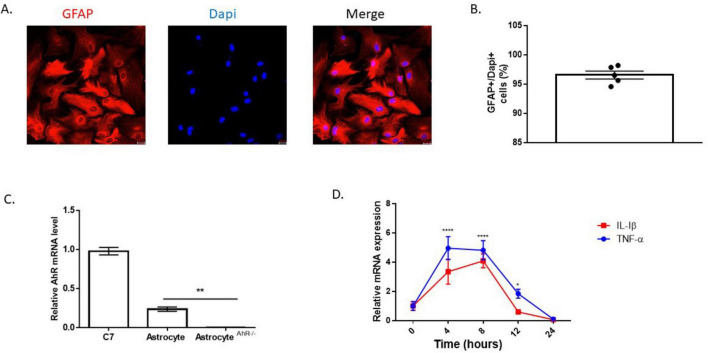
Time course induction of proinflammatory cytokines by Aβ (1-42). **(A)** Representative primary hippocampal astrocyte cell culture image stained with glial fibrillary acidic protein (GFAP) (red) and Dapi (blue). **(B)** Percentage of astrocytes present in astrocyte enriched culture (GFAP + DAPI + cells). Data are represented as mean ± S.E.M, *n* = 5 independent biological replicates. All images taken at 20× magnification from four different fields for each well. **(C)** mRNA levels of aryl hydrocarbon receptor (AhR) in C7 cells, C57BL6/J and global AhR knockout hippocampal astrocytes. Data are represented as mean ± S.E.M, *n* = 3 independent biological replicates. **P < 0.01 by One-way ANOVA with Tukey’s *post hoc* comparison. **(D)** mRNA expression for TNF-α and IL-1β following incubation with 5 μM Aβ (1-42) in astrocyte cell over a 24 h period shown. Data represent mean ± S.E.M, *n* = 4 independent biological replicates analyzed by One-way ANOVA with Tukey *post hoc* comparison. *P < 0.05, **P < 0.01, ****P < 0.0001 versus time zero.

To assess the inflammatory effects of Aβ (1-42), primary hippocampal astrocyte cultures were established. Purity of cultures was verified using the astrocyte marker, GFAP. Roughly 96% of the cells were positive for GFAP, and the morphology of the cultured astrocyte cells resembled the established *in vitro* cellular structure of astrocytes (Figures 1A, B). Furthermore, AhR was constitutively expressed in astrocyte cultures taken from C57BL6/J mice, but absent in cultures established from global AhRKO mice (Figure 1C). To examine the inflammatory effects of Aβ (1-42) on astrocyte cells, a time course induction of proinflammatory cytokine transcripts for TNF-α and IL-1β was determined. Inflammatory cytokine transcripts peaked 4–8 h after treatment with Aβ (1-42), suggesting these timepoints are optimal for experiments exploring inflammatory cytokines at the mRNA level (Figure 1D). Taken together, this result indicates that Aβ (1-42) induces inflammation in astrocytes in a time-dependent manner *in vitro*.

### 3.2 AhR activation attenuates reactive astrocyte phenotypic characteristics induced by Aβ (1-42)

**FIGURE 2:**
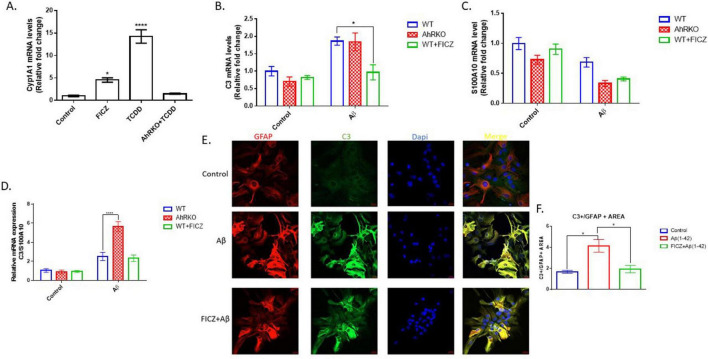
Effects of aryl hydrocarbon receptor (AhR) activation on Aβ (1-42) induced A1/A2 phenotype markers. **(A)** Fold change of mRNA levels relative to control expression of CYP1a1 in hippocampal astrocytes treated with, 6-Formylindolo[3,2-b] carbazole (FICZ) (250 nM), TCDD (10 nM) for 6 h. Data represent mean ± S.E.M, *n* = 3–4 independent cell culture, analyzed by One-way ANOVA with Tukey’s *post hoc* comparison. *P < 0.05, ****P < 0.0001 vs Control. **(B)** Fold change of mRNA levels relative to wide type (WT) expression of C3. **(C)** Fold change of mRNA levels relative to wide type (WT) expression of S100A10 expression. **(D)** Ratio of mRNA C3/mRNA S100A10 expression. Data represent mean ± S.E.M, *n* = 3–4 independent biological replicates analyzed by Two-way ANOVA with Tukey *post hoc* comparison. *P < 0.05, ****P < 0.0001. **(E)** Representative immunofluorescence stain of primary hippocampal astrocyte cell culture with GFAP (red), C3 (green) and Dapi (blue) after 24 h exposure with Aβ (1-42). **(F)** Quantification of astrocyte C3 area/GFAP area. Data represent mean ± S.E.M, *n* = 4 independent biological replicates. All images taken at 40× magnification from four different fields for each well.

**FIGURE 3:**
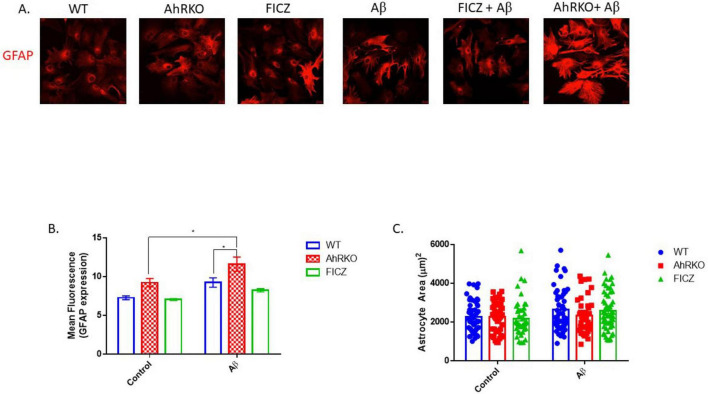
Aryl hydrocarbon receptor (AhR) deficiency exacerbates astrogliosis induced by Aβ. **(A)** Representative immunofluorescence stain of primary astrocyte cell culture with glial fibrillary acidic protein (GFAP) (red) after 24 h exposure with Aβ (1-42). **(B)** Quantification of astrocyte cells total area covered (μm^2^), total of 50 astrocyte cells per group from four independent cell culture. **(C)** Average mean GFAP fluorescent intensity quantification of astrocyte cells. Data represent mean ± S.E.M, *n* = 4 independent biological replicates. All images taken at 40× magnification from four different fields for each well. *P < 0.05 by Two-way ANOVA with Tukey’s *post hoc* comparison.

Astrogliosis is characterized by structural and molecular changes in astrocytes. The impacts of AhR activation on Aβ (1-42) -induced morphological changes, as well as induction of reactive astrocyte phenotype, were examined. To establish that AhR is activated effectively in astrocytes, the canonical downstream target *Cyp1a1* was assessed. AhR agonists, FICZ (250 nM) and TCDD (10 nM) increased *CYP1a1* transcript levels, in an AhR-dependent manner (Figure 2A). Subsequently, pretreatment of astrocytes with FICZ attenuated the reactive astrocyte marker induced by Aβ (1-42), as C3 gene expression and immunoreactivity were significantly reduced when compared to Aβ (1-42) only treatment group (Figures 2B, E, F); meanwhile, transcript levels of S100A10 remained unaffected by FICZ treatment (Figure 2C). C3 transcript levels induced by Aβ (1-42) were similar in Wild Type and AhRKO-derived astrocytes; however, the ratio of C3 to S100A10 transcripts was higher in AhRKO astrocytes after Aβ (1-42) treatment (Figure 2D). The deletion of AhR in astrocytes increased GFAP immunoreactivity induced by Aβ (1-42), which is another indicator for astrocyte reactivity (Figures 3A, B). Surprisingly, astrocyte size was unaffected by Aβ (1-42) treatment in both Wild Type and AhRKO-derived astrocytes (Figure 3C). Thus, our results suggest that activation of AhR by FICZ helps suppress Aβ (1-42) -induction of a reactive astrocyte phenotype.

### 3.3 AhR activation limits the astrocyte inflammatory response induced by Aβ (1-42) oligomers

**FIGURE 4:**
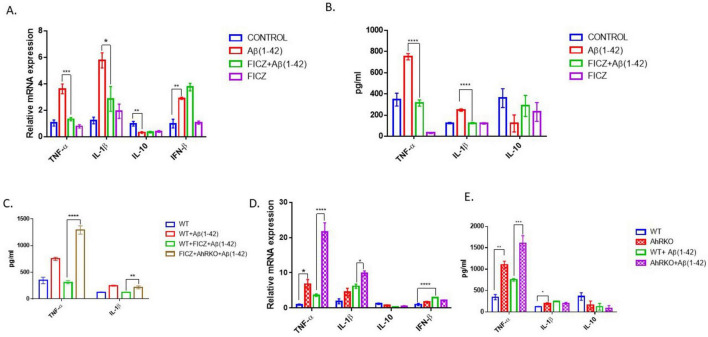
Aryl hydrocarbon receptor (AhR) activation suppress Aβ (1-42) induced inflammatory response in astrocyte. **(A)** Fold change of mRNA levels relative to control expression levels of inflammatory cytokines in hippocampal astrocytes pretreated with 6-Formylindolo[3,2-b] carbazole (FICZ) (250 nM) followed by Aβ (1-42) for 8 h. **(B,C)** Secretion level of inflammatory cytokines in primary astrocyte media after Aβ (1-42) exposure for 24 h. Data represent mean ± S.E.M, *n* = 3–4 independent biological replicates. *P < 0.05, **P < 0.01, ***P < 0.001, ****P < 0.0001 by One-way ANOVA with Tukey’s *post hoc* comparison. **(D)** Fold change of mRNA levels relative to wide type expression levels of inflammatory cytokines in AhR deficient astrocytes treated with by Aβ (1-42) for 8 h. **(E)** Secretion level of inflammatory cytokines in primary astrocyte media after Aβ (1-42) exposure for 24 h. Data represent mean ± S.E.M, *n* = 3–4 independent biological replicates. *P < 0.05, **P < 0.01, ****P < 0.0001 by Two-way ANOVA with Tukey’s *post hoc* comparison.

Cytokine profiling was conducted to better elucidate the effects of AhR signaling on Aβ-induced reactive astrocytes. Aβ markedly elevated transcripts levels and secretion of TNF-α and IL-1β; however, prior activation of AhR in astrocyte cell culture with FICZ [2 h before Aβ (1-42)] substantially reduced both transcript levels and secretion of TNF-α and IL-1β. Alternatively, the anti-inflammatory cytokines IL-10 and IFN-β were not significantly changed (Figures 4A, B). To assess whether the protective effects of FICZ on astrocytes are dependent on AhR activation, an analysis of proinflammatory cytokines (TNF-α and IL-1β) was conducted in astrocytes derived from AhRKO mice. In the absence of AhR, the protective effects of FICZ were diminished, as secreted levels of TNF-α and IL-1β were markedly increased in astrocytes lacking AhR, even in the presence of FICZ, suggesting that the effects of FICZ are dependent upon the presence of AhR (Figure 4C). Furthermore, AhR-deficient astrocytes exposed to Aβ (1-42) exhibited significant increases in the transcript levels of TNF-α and IL-1β when compared to Aβ (1-42) treated WT controls (Figure 4D). In a similar manner, the levels of secreted TNF-α were significantly elevated in astrocytes derived from AhRKO mice compared to Aβ (1-42) treated WT controls, whereas no differences were observed in IL-1β secretion levels (Figure 4E). The anti-inflammatory cytokine, IL-10 showed no significant changes; however, the increased transcript expression of IFN-β induced by Aβ (1-42) treatment was diminished in AhR-deficient astrocytes exposed to Aβ (1-42) (Figures 4D, E). These results imply that AhR signaling could be controlling astrocyte responses to inflammatory stimuli by inhibiting neurotoxic cytokines.

### 3.4 AhR Deletion in male mice exacerbates astrocyte inflammatory response to Aβ *in vivo*

**FIGURE 5:**
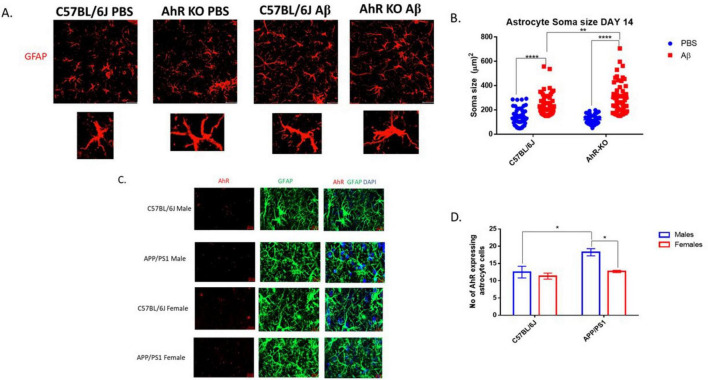
Aryl hydrocarbon receptor (AhR) Deletion in male mice exacerbates astrocyte inflammatory response to Aβ *in vivo*. **(A)** Representative immunofluorescence stain of astrocyte cells in the CA1 region of the hippocampus 14 days post-intrahippocampal injection with Aβ (1-42). **(B)** Quantification of astrocyte cell soma size (μm^2^), total of 60 astrocyte cells per group. Data represent mean ± S.E.M, *n* = 4 animals per group. **P < 0.01, ****P < 0.0001 by Two-way ANOVA with Tukey’s *post hoc* comparison. **(C)** Representative RNAscope stain of mRNA AhR (Red) and immunofluorescence stain of astrocyte with glial fibrillary acidic protein (GFAP) (green) in the DG region of the hippocampus. **(D)** Quantification of AhR^+^GFAP^+^ cells in the DG region of the hippocampus. Data represent mean ± S.E.M, *n* = 3–4 animals per group. *P < 0.05 by Two -way ANOVA with Tukey’s *post hoc* comparison. All images taken at 40× magnification.

To assess the effect of AhR deficiency on the inflammatory response of astrocytes in their native environment, structural alterations in male mice astrocytes were evaluated following CNS inflammation induced by Aβ *in vivo*. In this neuroinflammatory model, a substantial population of hippocampal astrocytes exhibits inflammatory-induced morphological alterations 14 days after intrahippocampal Aβ oligomer injection. Aβ generated an increase in astrocyte soma size compared to the control-treated group; furthermore, these structural alterations were exacerbated in astrocytes from male AhRKO mice, which had more hypertrophic characteristics than Aβ-treated wild-type controls (Figures 5A, B). To assess the significance of astrocyte AhR function in an AD mouse model that approximates AD progression, AhR levels were assessed in astrocytes of both males and females aged APP/PS1 mice. Twenty-months-old male APP/PS1 mice exhibited a significant increase in AhR transcript levels in astrocytes compared to age-matched WT controls (Figures 5C, D). Also, aged female APP/PS1 mice demonstrated reduced AhR transcript levels when compared to male APP/PS1 mice, suggesting a sex-specific difference in AhR expression within the astrocytes of aged APP/PS1 mice. Thus, these findings indicate that AhR deletion in mice increased astrocyte cell immunological response to inflammatory stimuli in an *in vivo* system, similar to results of the *in vitro* model.

## 4 Discussion

Astrocytes are a main component of the brain immune system, which engage in the inflammatory response. One mechanism by which Aβ accelerates AD progression is by activation of immunological responses in astrocytes (Garwood et al., 2011; Chen and Holtzman, 2022). This study investigated how AhR activation affects Aβ-induced astrocyte inflammation using both *in vitro* and *in vivo* models. AhR activation decreases induction of reactive astrocytes and reduces pro-inflammatory cytokine expression. On the contrary, AhR-deficiency in the *in vivo* Aβ injection paradigm enhanced astrocyte hypertrophy. These results offer new insights on the possible function of AhR signaling in astrocyte-mediated inflammation in AD progression.

Amyloid beta 42 exists in multiple forms; however, in this astrocyte culture model, the oligomeric form of Aβ (1-42) was employed to cause inflammation. This amyloid variant has been identified as the most neurotoxic form, and is commonly used to induce inflammation in both neuronal and glial cell cultures *in vitro*. Acute exposure to Aβ (1-42) oligomers significantly raised pro-inflammatory cytokines (TNF-α and IL-1β) transcript levels within 4–8 h post-treatment, indicating a rapid response of astrocytes to Aβ. The activation of these pro-inflammatory cytokines corresponds with earlier findings that highlighted the significance of astrocyte-mediated inflammatory response in AD progression (González-Reyes et al., 2017; Kim et al., 2024; Singh, 2022). Many immune cytokines have been associated with the initiation and pathogenesis of AD (Fu et al., 2016; Fu et al., 2019; Tan et al., 2014; Taylor et al., 2018). For example, proinflammatory cytokines, including TNF-α and IL-1β, produce neuroinflammation by activating astrocytes and microglia. Activation of AhR using an endogenous ligand (FICZ) markedly diminished the inflammatory response in primary astrocyte cultures, leading to a reduction in the production and secretion of TNF-α and IL-1β. These findings indicate that AhR signaling activation can help mitigate Aβ-induced neuroinflammatory pathways in astrocytes, which may be relevant to early phases of AD progression when astrocytes and microglia are primed to release proinflammatory cytokines in response to acute stimulation by Aβ.

To further support this observation, the suppressive effects of FICZ on astrocyte-mediated inflammation were AhR-dependent, as astrocytes derived from AhRKO mice exhibited an enhanced inflammatory response upon Aβ exposure, even in the presence of FICZ. Although AhR activation produced minimal impact on anti-inflammatory cytokines IL-10 and IFN-β, we speculate that this is due to more neurotoxic A1 astrocytes, which have been shown to release more proinflammatory cytokines than anti-inflammatory cytokines (Pan et al., 2024; Liddelow et al., 2017). Alternatively, it is also possible that the anti-inflammatory cytokine response, particularly IL-10 production, is stronger during the recovery phase than during the initiation phase of the astrocyte cell inflammatory response. A hallmark response of astrocytes during development of AD pathology is their switch from homeostatic state to a disease-associated astrocytes state (Ceyzériat et al., 2018; Habib et al., 2020). Although complement component 3 (C3), a prominent reactive astrocyte marker, has not been reported as a part of AD inflammatory progression and neurodegeneration, this marker is highly expressed in astrocytes of AD patients (Wu et al., 2019; Reichwald et al., 2009). Consistent with the elevation of reactive astrocyte markers seen in AD models, this study demonstrated that Aβ exposure significantly elevated C3 immunoreactivity and transcript levels. Particularly, AhR activation by FICZ suppressed Aβ-induced C3 expression, which further supports the idea that AhR activation by FICZ specifically suppresses reactive astrocytes in this *in vitro* model. Also, increased GFAP levels in the brain and blood are linked to astrogliosis, which results from the activation of astrocyte cells by amyloid beta plaques in AD (Bettcher et al., 2021; Sánchez-Juan et al., 2024; Kamphuis et al., 2014). AhR-deficient astrocytes had increased GFAP immunoreactivity after Aβ exposure, providing more evidence for AhR regulation of astrocyte immune responses, especially pertinent to AD pathology. AhR signaling may influence astrocyte polarization during the inflammatory response, suggesting that AhR mediates immunoplasticity by balancing derivation of astrocyte phenotypes. Although no significant morphological changes occurred following Aβ exposure in the monoculture model, the brief duration of Aβ exposure may have limited events to translational modifications of GFAP expression, without sufficient time to observe structural remodeling of astrocytes *in vitro*. The addition of serum in primary astrocyte culture methods is known to alter the morphology and metabolic functions of cultured astrocyte cells *in vitro* (Prah et al., 2019). In our model, astrocytes were cultured in serum-free media for 2 days prior to experimental Aβ exposure; however, the presence of serum in our cell culture model could also influence the GFAP immunoreactivity and morphological response of astrocyte cells to Aβ. Therefore, future studies accessing the impact of AhR deficiency in astrocyte cells with regards to GFAP immunoreactivity and morphologically changes in a serum free condition is also necessary. Under other conditions, AhR activation may mediate immunosuppressive effects through interactions with the STAT3 and Nrf2 pathways (Anwar-Mohamed, et al., 2014; Lin et al., 2022; Zhang et al., 2023). As this study did not examine the molecular mechanisms, it is essential to also clarify the particular molecular pathways by which AhR modulates astrocyte reactivity in response to Aβ.

To closely mimic inflammation processes observed in AD, intrahippocampal injection of Aβ was employed since this model have been described to be used as an acute experimental model of AD that can be used to study glia cell response to Aβ (Dufort-Gervais et al., 2020; Zussy et al., 2011; Ali et al., 2015; Malin et al., 2001). The Aβ injection model reinforced the *in vitro* findings, as AhR deletion exacerbated astrocyte soma hypertrophy in response to Aβ, further supporting the idea that AhR regulates astrocyte function and contributes to the changes in glial cell dynamics in AD. Given that this *in vivo* model represents an acute neuroinflammatory condition, comprehensive investigation is required to characterize AhR function in astrocyte cells within a more chronic AD mouse model, where inflammation and disease development may be assessed at various time intervals. Also, this *in vivo* investigation utilized global AhR knockout animals, allowing for potential confounding effects due to AhR deficiency in other immune cells within the brain, such as microglia. Consequently, it is essential to investigate the precise function of astrocyte AhR in response to CNS inflammatory stimuli, such as Aβ (1-42), while controlling for the confounding effects of AhR deficiency in other cell types within an *in vivo* context.

A sex-specific difference in AhR expression was noticed in astrocytes of aged APP/PS1 mice, with higher AhR expression observed in APP/PS1 male mice compared to females. This finding is particularly intriguing, given the well-documented sex differences in Alzheimer’s disease pathology, progression and neuroinflammation (Buckley, 2022; Arenaza-Urquijo et al., 2024; Zhu et al., 2021). There are well-documented sex differences reported in humans and several AD mice models, where females exhibit a higher risk of developing AD and often present with enhanced neuroinflammatory and Aβ-plaque burden; this finding, therefore, raises possibilities that the decreased AhR expression in female APP/PS1 mice astrocytes may be contributing to the sex-specific vulnerability related to AD pathology. This finding is interesting because AhR signaling is well-known to interact with estrogen receptor; and the decrease in estrogen levels in aging females may help explain the reduced AhR gene expression levels found in aged female APP/PS1 mice (Matthews and Gustafsson, 2006).

Overall, this study demonstrates the potential for AhR to act as a regulator of astrocyte-driven neuroinflammation in AD. AhR signaling represents a novel pathway to explore for potential new therapeutic approaches focused on minimizing astrocyte-mediated inflammation by suppressing reactive astrocyte and pro-inflammatory cytokine production. Future studies could explore the downstream molecular mechanisms underlying AhR immunosuppressive effects in AD models and assess the potential of other AhR ligands, especially since AhR receptor functions are ligand-specific.

## Data Availability

The raw data supporting the conclusions of this article will be made available by the authors, without undue reservation.

## Supplementary material

Supplementary Figure 1 AhR activation suppress LPS induced inflammatory response in astrocyte. **(A)** Fold change of mRNA levels relative to wide type expression levels of inflammatory cytokines in AhRdeficient astrocytes cells treated with LPS (250 ng/ml) for 24 hours. **(B)** Fold change of mRNA levels relativeto control expression levels of inflammatory cytokines in hippocampal astrocytes pretreated with FICZ(250 nM) followed by LPS. Data represent mean ± S.E.M, *n* = 4 independent biological replicates. **P* < 0.05,***P* < 0.01, *****P* < 0.0001 by Two-way ANOVA with Tukey’s post hoc comparison.
