# Supplementary material for: AhR regulation of amyloid beta-induced inflammation in astrocyte cells
Source: Front Cell Neurosci. 2025 Jul 8;19:1618209. doi: 10.3389/fncel.2025.1618209 (PMC12279702; doi:10.3389/fncel.2025.1618209)
Supplement: Supplementary Figure 1 — AhR activation suppress LPS induced inflammatory response in astrocyte. (A) Fold change of mRNA levels relative to wide type expression levels of inflammatory cytokines in AhRdeficient astrocytes cells treated with LPS (250 ng/ml) for 24 hours. (B) Fold change of mRNA levels relativeto control expression levels of inflammatory cytokines in hippocampal astrocytes pretreated with FICZ(250 nM) followed by LPS. Data represent mean ± S.E.M, n = 4 independent biological replicates. *P < 0.05,**P < 0.01, ****P < 0.0001 by Two-way ANOVA with Tukey’s post hoc comparison. [file Data_Sheet_1.pdf]

Supplementary figures

Table 1

Genes	Forward primer sequence	Reverse primer sequence
TNF-α	5’ -CCA CCA CGCTCT TCT GTCTAC-3”	5’- AGG GTC TGG GCCATA GAA CT -3’
IL-1β	5’-AGATGAAGGGCTGCTTCCAAA-3’	5’-GGAAGGTCCACGGGAAAGAC-3’
IL-10	5’- AGGCGCTGTCATCGATTTCT-3’	5’-ATGGCCTTGTAGACACCTTGG-3’
IFN-β	5’-CAGCTCCAAGAAAGGACGAAC-3’	5’-GGCAGTGTAACTCTTCTGCAT-3’
CYP1A1	5’-GTGCATCGGAGAGACCATTG-3’	5’-GGTAGGAGTCATATCCACCTT-3’
C3	5’-AGCTTCAGGGTCCCAGCTAC-3’	5’-GCTGGAATCTTGATGGAGACGC-3’
S100A10	5’-CCAGGTTTCGACAGACTCTTC-3’	5’-CCGTTCCATGAGCACTCTC-3’
GAPDH	5’-ATGGTGAAGGTCGGTGTGAAC-3’	5’-TGTAGTTGAGGTCAATGAAGG-3’
